# Diverging death risks: Mortality as a corollary of economic, social, cultural and person capital

**DOI:** 10.1016/j.ssmph.2024.101644

**Published:** 2024-02-29

**Authors:** Yuwei Qi, Sijmen A. Reijneveld, Josué Almansa, Sandra Brouwer, J. Cok Vrooman

**Affiliations:** a University of Groningen, University Medical Center Groningen, Department of Health Sciences, Groningen, the Netherlands; b Utrecht University, Department of Sociology/ICS, Utrecht, the Netherlands; c The Netherlands Institute for Social Research|SCP, the Netherlands

**Keywords:** Mortality, Socioeconomic factors, Social determinants of health, Capital

## Abstract

**Introduction:**

Diverging death risks are associated with a wide range of social factors, including not only education and income but also other economic and non-economic resources. The aim of this study was to assess the association of mortality risks with four types of resources: economic, social, cultural and person capital.

**Methods:**

We used data of 2,952 participants from the Disparities in the Netherlands survey and annual mortality data from Statistics Netherlands for the period 2014 to 2021. *Economic capital* was measured through education, income, occupation, home equity, and liquid assets. *Social capital* was measured by the strength of social ties, the size of the core discussion network, and access to people in resourceful positions; *cultural capital* by lifestyle, digital skills, and mastery of English, and *person capital* by self-rated health, impediments to climbing stairs, self-confidence, self-image, people’s appearance, and body mass index. To accommodate the fact that each capital was derived from several indicators, we used Partial Least Squares (PLS) Cox Regression.

**Results:**

In multiple regression, higher economic, cultural, and person capital were associated with lower mortality (hazard ratio, 0.77; 95% confidence interval [CI, 0.65 to 0.90], 0.77 [0.64–0.93] and 0.80; [0.70–0.92]), adjusted for all capital measures and sex.

**Conclusion:**

The finding that more economic, cultural and person capital is associated with lower mortality provides empirical support for an approach that uses a broad spectrum of capital measures - hitherto rarely included simultaneously in epidemiological research - in order to understand diverging death risks. By integrating sociological concepts, cohort data, and epidemiological research methods, our study highlights the need for further research on the interplay between different forms of resources in shaping health inequalities. In designing public health interventions, we advocate the adoption of a multidimensional capital-based framework for tackling social disparities in mortality.

## Highlights

• A wider set of capital measures - hitherto seldom included simultaneously in epidemiological research - can better explain divergent death risks.
• Exploring socioeconomic inequalities in mortality through a multidimensional lens may prove valuable, as it allows for a more comprehensive consideration of its dynamic and complex nature."

## 1 Background

Health inequalities are complex because they cannot be reduced to the linear impact of one or two factors that determine people’s physical and mental state over the entire life course. Both economic and non-economic resources have been found to be associated with variation in population health (Ehsan et al., 2019; Fancourt & Steptoe, 2019; Holt-Lunstad et al., 2010; Kawachi & Berkman, 2000; Shor & Roelfs, 2015; Torssander & Erikson, 2010). Studies on such resources that are typically connected to an individual’s social position, namely financial means and interpersonal support, often draw on theories of capital (Abel, 2008; Abel & Frohlich, 2012; Ahnquist et al., 2012; Angela, 2006; Dijkstra and Horstman, 2021, 2023; Ehsan et al., 2019; Kawachi, 2006; Khawaja & Mowafi, 2006; Pinxten & Lievens, 2014; Stephens, 2008; Veenstra & Patterson, 2012). Bridging these insights, this approach suggests a need to consider a spectrum of factors when studying health inequalities in general.

In a recent study we showed the empirical interconnectedness of different resources by building on a theoretical multidimensional framework developed by the Netherlands Institute for Social Research|SCP (Qi et al., 2022; Vrooman et al., 2014, 2023a). In the present study, we attempt to explore mortality as a manifestation of health inequalities through the lens of the four different forms of capital—economic, social, cultural, and person capital. In the SCP capital-based model (Vrooman et al., 2024), economic capital refers to people’s educational attainment and professional skills, labour market position, and income and wealth. In line with Becker's human capital theory (Becker, 2007), educational attainment is considered primarily as an economic resource, because it signifies the knowledge, skills and labour market qualifications people have acquired. Moreover, many educational systems explicitly aim to provide equal starting opportunities for their pupils and students; and historically, in late industrial society they have accomplished this task to some extent (Van de Werfhorst, 2014). Social capital consists of the resources that are embedded in the relationships individuals have with others. It refers to both one’s position in social networks, and the size and quality of those networks. In the SCP capital model, network assets may relate to financial and material support; the provision of information, influence or social credentials; emotional support; and the recognition of a person’s identity and group membership. Cultural capital concerns collective predispositions, expressive behaviours and attributes that mark social positions (language and communication; tastes, preferences and cultural knowledge; symbolic attributes such as formal titles and reputations). Although this demarcation largely builds on the work of Bourdieu who stressed distinction processes through participation in ‘high culture’, in the SCP capital-based model this theoretically also includes emerging forms (e.g., cultural omnivorism, ecologically responsible ways of living). Person capital comprises the (dis)advantages of an individual’s bodily and mental state. Theoretically, person capital has three subtypes: physical capital (bodily health and abilities), mental capital (psychological health and abilities) and aesthetic capital (individual traits that are attractive to others, such as beauty; the ‘right’ attitudes and behaviours). The first two elements are also known in the literature as 'health capital' or 'psychophysical capital' (Angela, 2006; Grossman, 1972). The third aspect concerns individual traits that are attractive to others, such as good looks or exhibiting certain attitudes and behaviours, e.g., charm (Sylvia & Giselinde, 2015; Van den Berg & Arts, 2019; Warhurst & Nickson, 2020). Following Pareto, who stressed that individuals may acquire social positions because they are physically, morally and intellectually different (Mackenbach, 2010), in the SCP capital-based model this concerns individual heterogeneity rather than ‘embodied’ forms of class-based cultural capital, as proposed by Bourdieu. This implies that person capital cannot be reduced to group characteristics: if two people are similar in their economic, social and cultural capital, they may end up quite differently in life if one is healthy and attractive, and the other is not.

Based on this theoretical multidimensional framework, SCP empirically identified a post-industrial class structure in the Netherlands, where each social class has a distinct combination of the four types of capital (Vrooman, Boelhouwer, Iedema, & van der Torre, 2023). In general, diverging death risks in the population may therefore not solely arise from individuals' economic capital but may also be influenced by their social, cultural, and person capital, because differential access to, and possession of, resources may have impact on people’s survival. These findings suggest that a broader set of capital measures—historically infrequently considered simultaneously in epidemiological research—may offer a more comprehensive explanation for diverging death risks in the Dutch population than a singular focus on economic resources. Theoretically a link with the various types of capital seems likely, but the empirical evidence is limited, except for the connection to economic capital (Pinxten & Lievens, 2014). It includes all kinds of material resources that could be used to acquire or maintain better health. Economic capital, partially captured by conventional socioeconomic status (SES) indicators like income, education, and occupational status, encompasses a variety of economic resources. Thus, different indicators of economic capital can reflect diverse aspects of an individual's economic resource base (Galobardes et al., 2006). Wealth, as one aspect of economic capital could be used to buffer the effects of income losses due to unemployment or illness, for instance by being able to afford better health treatments or live-in healthier environments (Kawachi, 2006). The association of social capital and cultural capital with mortality has been documented far less, although a few studies indicated an association between low social capital (Aida et al., 2011; Choi et al., 2014; Ehsan et al., 2019), and a lack of cultural capital with higher mortality (Abel, 2008; Bygren et al., 1996; Khawaja & Mowafi, 2006). In addition, Mackenbach suggested that personal characteristics can also partly account for health inequalities, and these inequalities are no less unfair than inequalities attributable to other economic or non-economic resources (Becker, 2007). Individuals may differ in a number of personal characteristics that promote good or bad health and might further contribute to inequalities in mortality. The distribution of individual characteristics underlying person capital may reflect individuals' past and present circumstances, as well as genetic variations (Harden, 2021; Mackenbach, 2010). Up till now, no studies have examined the effect of different types of resources on mortality simultaneously; their impact is typically assessed in a single-dimensional way (Fancourt & Steptoe, 2019; Holt-Lunstad et al., 2010; Kondo et al., 2009; Shor & Roelfs, 2015). The present study aims to address this gap, by investigating the independent effects of economic capital, social capital, cultural capital, and person capital on mortality during seven years follow-up.

## 2 Methods

### 2.1 Study population

We used data from the Disparities in the Netherlands (ViN) project that was initiated by the Netherlands Institute for Social Research|SCP (Vrooman et al., 2014). In the spring of 2014, a survey was held among nearly 3,000 Dutch people aged 18 and older. Statistics Netherlands (CBS) drew a representative sample from the population register, with an overrepresentation of the top and bottom 10% of the distribution of standardized disposable household income. Field work was conducted by I&O Research through a combination of an internet survey and written questionnaires, with a response rate of 43% (Vrooman et al., 2014). After these data had been gathered, CBS added variables relating to income, wealth, age, sex, household type and ethnic origin obtained from the national population register, tax authorities and benefit agencies.

The combined set of survey and register data was weighted by CBS to the national distributions of income, sex, age, ethnic origin, family composition, etc. Data are available in the public domain through the Data Archiving and Networked Services (DANS) of the Netherlands; however, for this project we had access to a more elaborate ViN-file that was provided by the Netherlands Institute for Social Research|SCP. Participants in the current study included 2,952 individuals. According to Dutch law, formal ethical assessment of the study protocol was not needed as the study did not involve an intervention and data from CBS were anonymized (based on guidance from the Central Committee on Research Involving Human Subjects (WMO) and the Dutch Personal Data Protection Act). CBS collects and produces population statistics, referred to as non-public microdata, for all registered Dutch citizens. Under strict conditions, these data are accessible for scientific research. The research board of CBS has reviewed and approved the study protocol (project number 8605). Furthermore, all data and analyses were checked on identifiability of individuals and organizations by an independent employee of CBS before releasing analyses for publication.

### 2.2 Measures

#### 2.2.1 Mortality

We extracted annual all-cause mortality data from CBS for the period 2014–2021, as reported on the death certificate and coded at the CBS. The year and month of death and causes of death were obtained for all the participants.

#### 2.2.2 Economic capital

Economic capital was measured through people’s highest successfully completed level of education, income levels, occupational prestige, and financial wealth. The response categories for level of education contained a detailed list of 17 Dutch types of education, running from elementary school (unfinished) to PhD, plus ‘other’ and ‘don’t know’, which were coded as missing. Income was measured by using registry data from the CBS. Standardized disposable household income was computed in percentiles. Occupational prestige was measured by the International Socio-Economic Index (ISEI08) (Ganzeboom, 2010). These prestige scores do not contain subjective status assessments, but instead represent a weighted score of each occupation’s earnings level and the required skill level. The ISEI effectively reflects the level of socioeconomic vulnerability (toward the lower end of the scale) or protection (toward the higher end of the scale) of contemporary occupations. Disposable household income was obtained through register data, as was the household’s wealth, indicated by liquid assets (in percentiles) and home equity (in percentiles). Liquid assets consisted of savings and investments, other movable and immovable property such as a second home, business-related assets such as company assets and substantial interests. Home equity was measured by the difference between a property's estimated annually price by the municipality and the remaining mortgage debt (Balestra & Oehler, 2023).

#### 2.2.3 Social capital

Social capital was measured by the strength of social ties (Granovetter, 1973), the size of the core discussion network (Burt, 1984; McPherson et al., 2006; Small, 2013), and access to people in resourceful positions. Strength of social ties was measured by the frequency of respondents’ contact with family, friends and neighbours. The score for strength of social ties ranged from 0 to 16 with higher scores indicating stronger social ties. Size of the core discussion network was measured by the number of people (not in the same household) with whom the respondents discuss important personal matters. This does not concern people who provide respondents with professional help, such as a general practitioner. The score for size of the core discussion network ranged from 0 to 4, with a higher score indicating a larger network. Access to people in resourceful positions was measured by asking if someone knows wealthy or influential people (a mayor or Member of Parliament; a doctor or lawyer; a director of a commercial company employing more than ten people; a high-ranking official; a professional musician, artist or writer) (Lin & Dumin, 1986; Lin et al., 2017, pp. 57–81; Van der Gaag et al., 2008). The score of access to people in resourceful positions ranged from 0 to 5, wither higher score indicating better access to people in resourceful positions.

#### 2.2.4 Cultural capital

Cultural capital was measured by lifestyle, basic digital skills, and mastery of English. Lifestyle consists of a scale for engagement in various cultural activities (foreign holidays, eating out for more than 100 euros per person and visits to 'higher' culture, such as a classical concerts, theatres or art museums). This lifestyle measure captured tastes and preferences in the Bourdieusian tradition (Vrooman et al., 2024). The score for lifestyle ranged from 0 to 8, with higher score indicating more engagement in cultural activities. Total score for basic digital skills ranged from 0 to 3, indicating the degree to which the respondents can work with a computer. Digital literacy, seen as an emerging type of cultural resource, was later conceptualized by Bourdieu as a component of 'technical capital,' representing a distinct subtype (Bourdieu, 2005; Emmison & Frow, 1998; Ollier-Malaterre et al., 2019). Mastery of English was measured on a continuous scale (ranging from 0 to 8), with a higher score indicating better language proficiency. This item was derived from the categorization outlined in the Common European Framework of Reference for Languages and streamlined into five distinct categories (Canagarajah, 2012; De Swaan, 2013).

#### 2.2.5 Person capital

Person capital was measured by self-rated health, impediments to climbing stairs, self-confidence, self-image, body mass index (BMI), and estimation of (own) appearance. Self-rated health was measured on a continuous scale, ranging from 1 (poor) to 5 (excellent) (Bowling, 2005). For impediments to climbing stairs, respondents were asked whether they are currently limited by health issues when climbing stairs, measured with a score ranging from 0 (not limited at all) to 2 (severely limited). This is a common, validated indicator of impairment in performing basic activities of daily living (Katz et al., 1970; Startzell et al., 2000). The mental aspect of person capital was covered by validated questions on self-confidence and self-image (Marsh & O'Neill, 1984; Van der Wal, 2004). Self-confidence was measured through the item ‘I have a lot of confidence’; the answers vary from 1 (strongly disagree) to 10 (strongly agree). Self-image was assessed by the statement ‘I have a negative image of myself”, once again on a 10-point scale. The aesthetic aspect of person capital was indicated by two proxies: BMI and estimation of one’s own appearance. BMI, associated with both physical and mental health as well as attractiveness, was calculated by dividing weight in kilograms by the square of height (in meters) (Brierley et al., 2016; Fletcher, 2014; Furnham et al., 2006). Appearance was measured by respondents’ responses on the items ‘I think my appearance is just fine’ and ‘Others think I look good’), both on a scale running from 1 (strongly disagree) to 10 (strongly agree). These newly created items for the 'Disparities in the Netherlands' survey were carefully and neutrally worded, deliberately avoiding terms like 'beauty' and 'ugliness’ (Vrooman et al., 2014).

#### 2.2.6 Covariates

Data on sex was obtained from CBS register data, which cover the entire Dutch population.

### 2.3 Theoretical assumptions

In our capital-based approach, each capital construct is considered formative, determined by the values of their corresponding capital indicators (Qi et al., 2022). This means that the capital indicators collectively contribute to the overall formation of their corresponding capital construct. Next, formative indicators are not interchangeable, the removal of a single capital indicator can alter the meaning of the capital construct it contributes to (Hair et al., 2021). For example, removing the health element from person capital transforms the proxies for attractiveness items into a different construct that can no longer be referred to as person capital. Moreover, the contribution of these indicators may vary. The contribution of each capital indicator on the construct score varies depending on the context; the weights assigned to the indicators are determined by the study population and the conceptual model. (Petter et al., 2007). While participants' responses on different indicators may align, it is also plausible that these indicators capture distinct aspects of resources, leading to only modest correlations - or even in different directions (Bollen & Diamantopoulos, 2017; Diamantopoulos & Winklhofer, 2001; Vrooman et al., 2024). Nonetheless, deriving these weights from empirical observations is more reliable and reflective of reality than making arbitrary, non-expert choices, such as assuming equal weights for all indicators. Finally, in our conceptual model, we posit that the influential factor on mortality is the capital construct, the combination of various resources, rather than individual capital indicators. This acknowledges that, for example, individuals may have low education levels yet possess a substantial economic capital, as exemplified by those with advantageous employment situations.

### 2.4 Analyses

We first analysed all variables by inspecting frequencies for categorical variables, and means and standard deviations for the continuous measures. Next, we assessed associations between the four types of capital and mortality using Partial Least Squares (PLS) Cox continuous-time proportional hazard models, with age as the time-scale. Since our objective was to estimate the effect of types of resources, each consisting of several elements, rather than examining the impact of individual elements of each capital, we employed Partial Least Squares (PLS). By doing so, we could accommodate the formative construct nature of each capital. Specifically, PLS captures the large part of the variance present in the observed indicators, rather than focusing solely on explaining associations with the indicators (Tenenhaus et al., 2005). The item BMI, as one of the indicators for person capital was rescaled, so that higher values indicate a better state. Prior to the rescaling, we have confirmed that there were no underweight individuals in the study sample. We then estimated the PLS Cox models in two steps. First, four crude models were estimated using PLS Cox regression for each capital measure, to obtain the indicators’ weights and their corresponding capital scores. Capital scores were estimated as weighted linear combinations of their indicators. The weights for the indicators were re-scaled to get a positive meaning of the capital scores (i.e., higher scores indicating having more resources). For each model, we tested and confirmed that the proportional hazards assumption was not violated. Second, all capital scores were added together in model 1 and subsequently adjusted for sex in model 2. Hazard ratio (HR) and 95% confidential intervals (95%CI) were calculated for all-cause mortality during the follow-up period. The analyses were performed using R version 3.6.2 with the plsRcox package (Bastien et al., 2014). Given that the plsRcox package did not accept missing values, we performed single missing data imputation using the R package MICE (Buuren & Groothuis-Oudshoorn, 2011). Without imputation, employing listwise deletion would reduce the sample size by about one-third, leaving a total of 1967 respondents with complete data for each capital indicator and mortality.

## 3 Results

Background characteristics of the sample and the variables used in the analyses are presented in Table 1. The mean age was 50.2 (SD 16.5), and the majority was female (50.5%). During the seven years follow-up period, 196 participants deceased. The larger number of missing observations are on occupation, resulting from self-employed, retired, and student.

**Table 1:** Background characteristics of the study sample (N = 2952)^a^.

		Mean (SD) or n (%) a	Missing (%)
**Demographics**			
***Sex***			
Male		1460 (49.5%)	0
Female		1492 (50.5%)	0
***Age***	(in years)	50.2 (16.5)	0
**Constructs and corresponding indicators**			
***Economic capital***			
Education a		9.0 (4.92)	4.2
Occupation b		50.2 (21.3)	14.6
Income c		56.9 (29.6)	5.7
Home equity c		50.0 (28.9)	0.2
Liquid assets c		50.0 (28.6)	0
***Social capital***			
Strength of social ties^d^		4.7 (3.2)	1.0
Size of the core discussion network e		2.9 (1.2)	8.0
Access to people in resourceful positions f		1.9 (1.5)	8.8
***Cultural capital***			
Lifestyle g		1.3 (0.8)	0.9
Basic digital skills h		2.2 (1.1)	1.7
Mastery of English i		4.3 (1.7)	0.9
***Person capital***			
Self-rated health j		3.3 (0.9)	0.5
Impediments to climbing stairs k		7.4 (1.7)	0.4
Self-confidence l		6.8 (1.8)	2.4
Self-image m		7.8 (2.2)	4.1
Estimation of (own) appearance n		6.8 (1.8)	2.2
BMI o		25.8 (9.4)	1.2

*All variables were treated as continuous in the Cox-regression models. Higher score indicates a better situation.

a Education ranges from 1 to 17.

b Occupation was measured by the International Socio-Economic Index (ISEI08).

c Income, Home equity, Liquid assets were in percentiles.

d Strength of social ties ranges from 0 to 16.

e Size of the core discussion network ranges from 0 to 4.

f Access to people in resourceful positions ranges from 0 to 5.

g Lifestyle ranges from 0 to 8.

h Basic digital skills ranges from 0 to 3.

i Mastery of English ranges from 0 to 8.

j Self-rated health ranges from 1 to 5.

k BMI was rescaled, where a higher score indicates a healthier BMI.

l Impediments to climbing stairs ranges from 0 to 2.

m Self-confidence ranges from 1 to 10.

n Self-image ranges from 1 to 10.

o Appearance ranged from 1 to 10.

The associations between economic, social, cultural, person capital and mortality are presented in Table 2. Simple regression analyses showed higher economic capital, cultural capital and person capital to be associated with lower mortality. No statistically significant relation between social capital and mortality was found. When all four types of capital were simultaneously included in Model 1, higher economic, cultural and person capital were associated with lower mortality. Controlling further for sex in Model 2 did not change these associations.

**Table 2:** Hazard ratios (HR) and their 95% confidence intervals (95% CIs) of all-cause mortality by economic, social, cultural and person capital: crude and mutually adjusted hazard ratios, and hazard ratios after additional adjustment for sex.

	Crude HR (95%CI)	Adjusted HR (95%CI), Model 1^a^	Adjusted HR (95%CI), Model 2^b^
Economic capital	0.79 [0.68, 0.92]	0.78 [0.67, 0.92]	0.77 [0.65, 0.90]
Social capital	0.94 [0.78, 1.13]	1.03 [0.85, 1.26]	1.04 [0.85, 1.27]
Cultural capital	0.82 [0.70, 0.96]	0.72 [0.61, 0.84]	0.77 [0.64, 0.93]
Person capital	0.80 [0.71, 0.91]	0.80 [0.70, 0.92]	0.80 [0.70, 0.92]

a Model 1 = Economic capital + Social capital + Cultural capital + Person capital.

b Model 2 = Model l + sex.

The weights of indicators, as shown in Table 3, indicate the degree to which these contribute to the effects of their corresponding capital measures. Most of the indicators of capital measures (for which higher values are more favourable) had positive weights. This implies that an increase in any of these elements will result in a higher capital score. Occupation and home equity were the most influential elements of economic capital. For cultural capital, basic digital skills is the most important variable. Self-rated health and impediments to climbing stairs, together with estimation of one’s appearance were the most important aspects of person capital. To see whether assuming equal weights for each capital indicator may result in different findings compared to the PLS method, we performed additional sensitivity analysis by using the Z scores of each capital indicator and added the Z scores up as a rather arbitrary total score of the four capital measures (Appendix 1). In comparison with the PLS method, assuming equal weights for each capital indicators resulted in statistically non-significant Cox models, except for person capital.

**Table 3:** Weights of indicators for economic, social, cultural and person capital.

Indicator	Economic capital	Social capital	Cultural capital	Person capital
Education	0.22			
Occupation	0.64			
Income	0.16			
Home equity	0.70			
Liquid assets	0.14			

Strength of social ties		−0.30		
Size of core discussion network		0.19		
Access to people in resourceful positions		0.93		

Lifestyle			0.15	
Basic digital skills			−0.97	
Mastery of English			0.20	

Self-rated health				0.72
Impediments to climbing stairs				0.40
Self-confidence				0.04
Self-image				0.28
Estimation of (own) appearance				0.42
BMI				0.27

## 4 Discussion

In this study we found higher economic, cultural and person capital to be associated with lower mortality. These findings contribute to the growing literature supporting a multidimensional concept of inequalities in health and suggest that both economic and non-economic resources contribute independently to divergent death risks.

Our main finding, concerning the independent effect of economic, cultural and person capital on mortality indicates that not only economic resources, but also other forms of socially valued capitals are important in understanding the distribution of life chances in contemporary society. Distinct effects of various forms of capital also suggest that each capital may be important in the delineation of social disparities. By operationalizing each capital construct as formative, we examined the four forms of capital as independent predictors of mortality, acknowledging that these capital constructs are shaped by their corresponding indicators. Consequently, it is important not to view each capital indicator in isolation but rather as part of a broader combination that encompasses comprehensive measures of inequalities. Each indicator makes a unique contribution to the formation of the corresponding capital construct. Taking the formation of economic capital as an example, income is generally a more immediate economic resource, reflecting a person's earning capacity within a specific time frame. Wealth, on the other hand, represents a person's accumulated assets and savings over time. While income is an essential factor in day-to-day well-being, wealth and health have far-reaching consequences on an individual's long-term economic stability and overall quality of life. Someone with a higher income but lower education or occupation level can possess the same economic capital as someone with a somewhat lower income but a higher level of education. The sensitivity analysis further confirmed the need to consider various capital indicators as having its specific contribution to forming the capital. Consistent with the literature, the present study suggested that having more economic capital is generally associated with lower mortality (Katikireddi et al., 2020; Kondo et al., 2009; Mackenbach & de Jong, 2018). Our results further implies that wealth is an important element in bringing this about; and that it is important to consider multiple aspects of economic resource disparities to uncover potential class-related mechanisms that contribute to inequalities in mortality. While educational level and occupation are widely used, these variables are indirectly related to an individual’s material resources. Furthermore, they may be less relevant for specific age groups (Katikireddi et al., 2020). Wealth is related to income, but also implies a stock of assets (e.g., home equity) that may serve as a beneficial resource (Baum, 2005; Katikireddi et al., 2020). In the ViN sample, we observed a strong relationship between home equity and economic capital. While home equity is influenced by income, the wealth accumulated over one's lifetime may exert a more significant impact on health production. A study conducted in Spain supports this idea, demonstrating that housing equity, particularly in the form of housing assets, has a more substantial influence on health and disability in old age than income alone (Costa-Font, 2008). Home equity is indeed frequently missed in measuring economic conditions, whereas it constitutes a major economic resource (Swope & Hernández, 2019). Our findings suggest a need to shift the current discourse to place greater emphasis on a broader understanding of economic conditions that influence health.

We found that social capital did not have an effect on mortality, a finding that is not consistent with previous literature in which higher social capital was associated with reduced mortality (Rodgers et al., 2019). Nonetheless, Muennig et al. also found that social capital (measured by visiting friends and family) was not consistently predictive of intermediate measures of mortality (Muennig et al., 2013). Similarly, Choi et al. did not find strong evidence of social capital affecting all-cause mortality in their systematic review of prospective studies, although there was limited evidence for some specific dimensions of social capital (Choi et al., 2014). And finally, a larger or stronger social network may be beneficial to health, but if e.g., there are many unhealthy norms within such a social network it may also be detrimental to mortality risks. These aspects of social capital might not have captured its impact on health outcomes fully in the ViN sample. The most promising indicator of social capital is perhaps the one concerning "access to people in resourceful positions", as this might be indicative of health-beneficial social capital. We have therefore performed a sensitivity analysis that only looks at the association between access to people in resourceful positions and mortality, however, this aspect did not exhibit a significant association with mortality (not shown in the results, OR [CIs]: 1.02 [0.85, 1.12]). On the other hand, ‘a doctor or lawyer’ was only one of the five influential professions asked about in the survey. Our measurement may therefore not have been specific enough, and it measures access: we do not know what help the doctors they know could actually provide to individual respondents, and whether they would be willing to do so. Future research is needed to investigate whether the impact of social capital is masked or modified by measurement issues and the presence of other capital types.

The results of this study also suggest that having more cultural capital is associated with lower mortality. Previous longitudinal studies have shown that active participation in organized groups and associations, and engagement in leisure activity are important predictors of survival (Konlaan et al., 2000, 2002). The present study confirms these findings. The indicators utilized in our study may exhibit varying causal orderings in relation to mortality. For instance, activities like attending concerts inherently involve a certain level of economic capital. Methodologically, this suggests that they could act as mediators in the relationship between economic resources and mortality. By incorporating these variables into the same regression models, there is a likelihood that the impact of economic resources on mortality may be substantially attenuated. Nevertheless, it is important to highlight that the cultural and taste aspects associated with these indicators continue to exert influence in the relationship between cultural capital and mortality, as evidenced by their statistically significant effects. While acknowledging these complexities, it's essential to note that in this paper, we specifically focus on a partial aspect of the overall association, and the potential mediating roles of certain indicators could be explored in future research to provide a more comprehensive understanding of the relationships at play. Notably, we observed that basic digital skills was associated with cultural capital in an unexpected direction in this study population, considering that digital skills have the potential to impact health outcomes (Paasche-Orlow & Wolf, 2007). We have performed univariate analysis with only this indicator and mortality. The result (not shown in the manuscript) suggested scoring higher scores on basic digital skills was associated increased mortality, the underlying reasons for this discrepancy remain to be explored.

Next, this study showed having more person capital to be protective against mortality. Individuals with more person capital normally tend to grow old healthier and live longer. And if at some point, he or she does become ill, their circumstances and the consequences may often be more favourable (Vrooman et al., 2014). The addition of person capital is part of the theoretical framework undertaken in the present study as an attempt to incorporate “embodied” inequalities as part of the inequalities in mortality. Indicators such as self-rated health and impediment to climbing stairs were treated as indicators of person capital while these indicators are associated with both health itself and mortality. Although the empirical evidence on the mechanisms of social causation and health selection is mixed, recent systematic reviews indicate that both factors play a role in linking social position with health inequalities (Kröger et al., 2015; Mackenbach, 2012). The dominance of one over the other depends on the life stages and aspects of socio-economic status being examined. This highlights the importance of rethinking the role of health in shaping the inequities as studied (Grundy & Sloggett, 2003; Williamson & Carr, 2009). This conceptualization of health as resources aligns with Mackenbach's (Mackenbach, 2019) assertion that health plays a pivotal role in determining individuals' social positions. According to Mackenbach, social inequality not only causes health disparities but health can also, in turn, exacerbate social distinctions, creating vicious cycles. Additionally, the idea that health serves as a stock of biopsychosocial resources, enabling individuals to participate in society, implies health as a valuable asset (Williamson & Carr, 2009). Person capital is partly about initial health, but also about someone’s appearance and attractiveness (Qi et al., 2022; Vrooman et al., 2024). The distribution of these attributes may reflect people’s past and present circumstances, but can also be rooted in genetic differences between individuals (Conley & Fletcher, 2017; Harden, 2021; Vrooman et al., 2024). While it may be self-evident that indicators such as SRH are related to mortality, removing health elements of person capital in the analysis still resulted in an HR of 0.79 [0.69, 0.91] (Supplementary Table 3), showing that this further health element is not the dominant reason why person capital is associated with mortality. It is worth mentioning that removing the health elements of person capital will change the definition of person capital due to its formative conceptualization. Proxies for appearance and attractiveness, as measured by estimation of (own) appearance, showed a weight of 0.42 for person capital, which is approximately the same weight as impediments to climbing stairs. This result suggested that for our study sample, the appearance aspect of person capital is at least as important as the health aspect of person capital when it comes to the effect of various forms of capital on mortality. Differences related to looks go beyond money and affect how we make friends, or whether people are likely to help us (Anyzova et al., 2018; Glass et al., 2010; Hamermesh, 2011; Jaeger, 2011). The concept of attractiveness extends well beyond conventional facial and bodily features, encompassing a wide array of attributes such as skin colour, muscularity, smell, tone of voice, dental condition, grooming choices, appeal, and psychological traits like charm (Sarpila et al., 2021). Various professions are now requiring what is termed 'aesthetic labour,' emphasizing the need for individuals to project specific looks and behaviours, thereby intertwining appearance with occupational success (Warhurst & Nickson, 2020). In fact, the concept of attractiveness and its counterpart, ugliness, are not merely personal attributes but seem to be ingrained constitutive elements of the broader class structure, shaping social interactions, professional opportunities, and broader societal perceptions (Hamermesh, 2011; Vrooman et al., 2024). The finding that economic and person capital each independently influence mortality relates closely to the ongoing debate between two key hypotheses on health inequalities: health selection and fundamental causes. "Health selection" posits that a person's health influences their SES with healthier individuals attaining higher status and those with poorer health ending up in lower status roles (Foverskov & Holm, 2016). In contrast, "fundamental causes " argues that higher SES provides health benefits not available in to people in lower SES groups (Phelan et al., 2004). This results in a health gradient shaped by the unequal distribution of resources that vary across social strata. In our understanding, both of these hypotheses interpret the relationship between social position and health as basically linear and unidirectional. Following a capital-based approach, these two hypotheses became not mutually exclusive, and may operate simultaneously and reinforce each other. The inequalities in mortality being documented in the present study are the results of the interaction and accumulation of both economic and non-economic resources, including health itself. Following this perspective, the relationship between specific economic capital (as reflected in SES) and health would not be assumed to be unidirectional and linear across the social gradient, but to function through multiple pathways operating at several different levels (Oversveen et al., 2017). Future research is necessary to delve deeper into the interactions between the four forms of capital, to better understand how these complex dynamics contribute to health inequalities.

We recognize that single-dimensional measures of resources alone do not fully capture the complexities of health inequalities and their effects on mortality. Our findings suggest that the independent effects of economic, cultural, and person capital play a significant role in shaping inequalities and their impact on mortality. It is through the integration of these diverse capital dimensions that we can better comprehend the complex interplay of resources and their influence on health outcomes. Therefore, we emphasize the importance of examining multiple capital indicators collectively to gain a more nuanced understanding of the social determinants of mortality. One of the strengths of this study was that it used nationally representative data with a wide range of variables indicating various forms of capital. Moreover, our study benefited from the availability of mortality data for a follow-up period of seven years. Although all the capital indicators were measured only at baseline, the study results give objective information about the relationship between different types of capital and mortality. However, our study also has limitations. First, most capital indicators were self-reported; and although many of these were based on validated instruments or refer to forms of behaviour (e.g., museum visits) that are easy to quantify by respondents, this could have introduced some measurement bias. Second, some of the ordinal indicators (e.g., education, basic digital skills, impediments to climbing stairs) were handled as if they were continuous, due to the fact that PLS Cox Regression requires continuous variables in order to compute weighted scores. Third, we focused on all-cause mortality, which leaves to be assessed whether associations differ for specific sets of cause of death. Fourth, although broad assessments of capital are better predictors of mortality, how to translate this to real practice may be not instantaneously clear. For instance, one unit increase in economic score does not necessarily mean one unit increase in income or occupation. Rather, it is one unit increase of the combined economic resources that correspond to economic capital. Nonetheless, these capital scores can be used to build synthetic, high-level descriptive indicators of inequalities as they indicate the general direction in which specific types of resources might impact mortality or other health outcomes. It can also be used as a “control” variable to account for the possible confounding effect of pre-existing differences on the outcome of interest. Finally, the effect of the four forms of capital is context-dependent, which might limit the generalizability of our findings to some extent. For a given set of capital indicators, their weights are entirely structural (Howell et al., 2007). In other words, these weights of each indicator relative to its capital construct rely on the specific context in which they are estimated, i.e., the risk of death within the ViN population. According to Heise (Heise, 1972), a formatively measured construct is more than just a compilation of its individual measures. Instead, it represents the composite that most effectively predicts the dependent variable in the analysis. Hence, the meaning of the capital construct is equally shaped by mortality as it is by its indicators.

## 5 Conclusions

To conclude, our findings imply that analysing different forms of capital simultaneously can have added value. This study provides empirical support for using a broad spectrum of capital measures in studying divergent death risks. Future research should examine different types of interplay between the forms of capital in observational-trial settings. In practical terms, the concept of capital is of particular importance for programs and policies that are based on a simplistic approach to reducing inequalities in mortality. It is common to focus on the accumulation of socio-economic disadvantage over the life course in explaining diverging death risks. In this tradition, educational attainment translates into occupational career and income position, setting people on different health trajectories that culminate in mortality disparities. While the impact of economic capital on mortality persists in our analysis (with the highest weights for occupational prestige and housing equity), cultural and person capital turn out to be relevant as well. This suggests that mortality inequalities are based on a complicated empirical nexus of different types of resources, which means that they are likely to be best tackled by interventions that are not limited to classic socio-economic health disparities. Taking cultural capital as an example, the results produced by our indicators suggest that differences in ‘what one belongs to’ play a role in mortality inequality. This is not to say that the latter will disappear as a matter of course if social differences in theatre or restaurant attendance are reduced, for example by subsidising such cultural participation for people at the bottom of society. However, there are likely to be benefits from raising awareness of the impact of people's cultural capital on their interactions with health professionals (Willems et al., 2005), and from developing information, protocols and facilities that enable culturally sensitive interaction with patients in the final stages of life (Cain et al., 2018).Such interventions could be studied in large observational studies that include measurements of various health outcomes and the wide range of capital indicators identified in this study.

## Declaration of interest

None.

## Funding

This research did not receive any specific grant from funding agencies in the public, commercial, or not-for-profit sectors.

## Ethics and data sharing statement

The Disparities in the Netherlands (ViN) survey serves as an empirical basis for the Netherlands Institute for Social Research (SCP) report, which was published at the end of 2014. In this survey, SCP collaborated with Statistics Netherlands (CBS) by which the address details of the prospective participants for the survey were provided. The data provided by the participants were automatically combined with population registry data from CBS. The research population consists of residents of the Netherlands. According to Dutch law, formal ethical assessment of the study protocol was not needed as the study did not involve an intervention and data from CBS were anonymized (based on guidance from the Central Committee on Research Involving Human Subjects (WMO) and the Dutch Personal Data Protection Act). CBS collects and produces population statistics, referred to as non-public microdata, for all registered Dutch citizens. Under strict conditions, these data are accessible for scientific research. The research board of CBS has reviewed and approved the study protocol (project number 8605). Furthermore, all data and analyses were checked on identifiability of individuals and organizations by an independent employee of CBS before releasing analyses for publication.

All questionnaire data have been deposited by SCP/CBS at the Data Archiving and Network Services (DANS). For legitimate researchers, these are therefore available in the public domain.

## CRediT authorship contribution statement

**Yuwei Qi:** Writing – review & editing, Writing – original draft, Methodology, Formal analysis, Data curation, Conceptualization. **Sijmen A. Reijneveld:** Writing – review & editing, Writing – original draft, Supervision, Project administration, Methodology, Conceptualization. **Josué Almansa:** Writing – review & editing, Writing – original draft, Methodology, Formal analysis, Data curation. **Sandra Brouwer:** Writing – review & editing, Writing – original draft, Supervision, Project administration, Methodology. **J. Cok Vrooman:** Writing – review & editing, Writing – original draft, Supervision, Project administration, Methodology, Conceptualization.

## Figures and Tables

**Table:** 

	Crude HR (95%CI)	HR (95%CI) Model 3^c^	HR (95%CI) Model 4^d^
Economic capital	1.07 [0.88, 1.00]	0.92 [0.87, 1.00]	0.92 [0.85, 0.98]
Social capital	0.94 [0.86, 1.00]	0.93 [0.84, 1.03]	0.92. [0.83, 1.02]
Cultural capital	1.02 [0.95, 1.10]	1.10 [1.00, 1.21]	1.10 [1.00, 1.20]
Person capital	0.94 [0.90, 0.98]	0.93 [0.88, 0.99]	0.92 [0.87, 0.97]

^c^ Model 3 = Economic capital + Social capital + Cultural capital + Person capital.

Each capital score was the sum of the corresponding indicators’ Z scores.

^d^ Model 4 = Model 3 + sex.

## Data Availability

The authors do not have permission to share data.

## Abbreviations

SES: Socioeconomic status
BMI: Body Mass Index
PLS: Partial Least Squares
HR: Hazard Ratios
CIs: Confidence Intervals
