# Supplementary material for: Diverging death risks: Mortality as a corollary of economic, social, cultural and person capital
Source: SSM Popul Health. 2024 Feb 29;25:101644. doi: 10.1016/j.ssmph.2024.101644 (PMC10937154; doi:10.1016/j.ssmph.2024.101644)
Supplement: Multimedia component 1 [file mmc1.docx]

Supplementary Table 1: Correlations between the capital indicators

|  | Education | Occupation | Income | Home equity | Liquid assets | Strength of social ties | Size of the core discussion network | Access to people in resourceful positions | Lifestyle | Basic digital skills | Mastery of English | Self-rated health | Impediments to climbing stairs | Self-confidence | Self-image | Estimation of (own) appearance n | BMI o |
| --- | --- | --- | --- | --- | --- | --- | --- | --- | --- | --- | --- | --- | --- | --- | --- | --- | --- |
| Education | 1 |  |  |  |  |  |  |  |  |  |  |  |  |  |  |  |  |
| Occupation | 0.69 | 1 |  |  |  |  |  |  |  |  |  |  |  |  |  |  |  |
| Income | 0.46 | 0.50 | 1 |  |  |  |  |  |  |  |  |  |  |  |  |  |  |
| Home equity | 0.27 | 0.30 | 0.28 | 1 |  |  |  |  |  |  |  |  |  |  |  |  |  |
| Liquid assets | 0.03 | 0.09 | 0.05 | 0.41 | 1 |  |  |  |  |  |  |  |  |  |  |  |  |
| Strength of social ties | 0.03 | 0 | 0.10 | -0.03 | -0.04 | 1 |  |  |  |  |  |  |  |  |  |  |  |
| Size of the core discussion network | 0.26 | 0.21 | 0.07 | 0.06 | 0 | -0.17 | 1 |  |  |  |  |  |  |  |  |  |  |
| Access to people in resourceful positions | 0.36 | 0.30 | 0.21 | 0.19 | 0.05 | -0.14 | 0.26 | 1 |  |  |  |  |  |  |  |  |  |
| Lifestyle | 0.02 | 0.06 | 0.02 | 0.08 | 0.11 | -0.01 | 0.01 | 0.02 | 1 |  |  |  |  |  |  |  |  |
| Basic digital skills | 0.41 | 0.32 | 0.29 | 0.05 | -0.09 | 0.08 | 0.18 | 0.25 | -0.09 | 1 |  |  |  |  |  |  |  |
| Mastery of English | 0.57 | 0.46 | 0.29 | 0.13 | -0.05 | 0 | 0.29 | 0.35 | -0.02 | 0.51 | 1 |  |  |  |  |  |  |
| Self-rated health | 0.26 | 0.17 | 0.140 | 0.16 | -0.01 | -0.01 | 0.12 | 0.18 | -0.05 | 0.24 | 0.27 | 1 |  |  |  |  |  |
| Impediments to climbing stairs | 0.23 | 0.15 | 0.16 | 0.12 | 0.02 | 0.04 | 0.10 | 0.13 | -0.06 | 0.26 | 0.25 | 0.48 | 1 |  |  |  |  |
| Self-confidence | 0.11 | 0.10 | 0.16 | 0.10 | 0.04 | -0.07 | 0.06 | 0.17 | 0.02 | 0.10 | 0.14 | 0.21 | 0.10 | 1 |  |  |  |
| Self-image | 0.06 | 0.08 | 0.14 | 0.10 | 0.06 | -0.06 | 0.01 | 0.07 | 0.04 | 0.05 | 0.08 | 0.17 | 0.07 | 0.51 | 1 |  |  |
| Estimation of (own) appearance n | 0.05 | 0.06 | 0.03 | 0.06 | -0.02 | -0.06 | 0.10 | 0.11 | -0.01 | 0.01 | 0.07 | 0.20 | 0.11 | 0.39 | 0.23 | 1 |  |
| BMI o | 0.12 | 0.07 | 0.03 | 0.06 | 0.02 | 0.03 | 0.05 | 0.05 | 0.04 | 0.07 | 0.11 | 0.09 | 0.11 | 0.02 | 0..01 | 0.05 | 1 |

Supplementary Table 2: Correlations between the four forms of capital score

|  | Economic capital | Social capital | Cultural capital | Person capital |
| --- | --- | --- | --- | --- |
| Economic capital | 1 |  |  |  |
| Social capital | 0.34 | 1 |  |  |
| Cultural capital | -0.16 | -0.18 | 1 |  |
| Person capital | 0.27 | 0.21 | -0.20 | 1 |

Supplementary Table 3 Hazard ratios (HR) and their 95% confidence intervals (95% CIs) of all-cause mortality by economic, social, cultural and non-health elements of person capital *: crude and mutually adjusted hazard ratios, and hazard ratios after additional adjustment for sex.

|  | **Crude HR (95%CI)** | **Adjusted HR (95%CI), Model 1^a^** | **Adjusted HR (95%CI), Model 2^b^** |
| --- | --- | --- | --- |
| Economic capital | 0.79  [0.68, 0.92] | 0.79  [0.68, 0.95] | 0.78  [0.67, 0.92] |
| Social capital | 0.94  [0.78, 1.13] | 1.04  [0.84, 1.27] | 1.04  [0.85, 1.27] |
| Cultural capital | 0.82  [0.70, 0.96] | 0.78  [0.66, 0.92] | 0.86  [0.72, 1.03] |
| Non-health elements of person capital* | 0.80  [0.69, 0.92] | 0.80  [0.69, 0.93] | 0.79  [0.69, 0.91] |

*Health related item, i.e. self-rated health, impediments to climbing stairs were removed; additionally, BMI was removed.

Supplementary Table 4. Weights of indicators for economic, social, cultural and non-health elements of person capital*

| **Indicator** | **Economic capital** | **Social capital** | **Cultural capital** | **Person capital** |
| --- | --- | --- | --- | --- |
| Education | 0.22 |  |  |  |
| Occupation | 0.64 |  |  |  |
| Income | 0.16 |  |  |  |
| Home equity | 0.70 |  |  |  |
| Liquid assets | 0.14 |  |  |  |
|  |  |  |  |  |
| Strength of social ties |  | -0.30 |  |  |
| Size of core discussion network |  | 0.19 |  |  |
| Access to people in resourceful positions |  | 0.93 |  |  |
|  |  |  |  |  |
| Lifestyle |  |  | 0.15 |  |
| Basic digital skills |  |  | -0.97 |  |
| Mastery of English |  |  | 0.20 |  |
|  |  |  |  |  |
| Self-confidence |  |  |  | 0.12 |
| Self-image |  |  |  | 0.63 |
| Estimation of (own) appearance |  |  |  | 0.77 |

* Health related item, i.e. self-rated health, impediments to climbing stairs were removed; additionally, BMI was removed.
